# 4D-QSAR: Perspectives in Drug Design

**DOI:** 10.3390/molecules15053281

**Published:** 2010-05-04

**Authors:** Carolina H. Andrade, Kerly F. M. Pasqualoto, Elizabeth I. Ferreira, Anton J. Hopfinger

**Affiliations:** 1 Laboratory of Molecular Modeling, Faculty of Pharmacy, Federal University of Goiás, 1ª Av. c/ Praça Universitária, S/N., Goiânia, Goiás, 74605-220, Brazil; 2 College of Pharmacy, MSC09 5360, 1 University of New Mexico, Albuquerque, New Mexico 87131-0001, USA; E-Mail: hopfingr@unm.edu (A.J.H.); 3 Faculty of Pharmaceutical Sciences, Av. Prof. Lineu Prestes, 580, University of Sao Paulo, Sao Paulo, 05508-900, Brazil; E-Mails: kerly@usp.br (K.F.M.P.); hajudan@usp.br (E.I.F.); 4 The Chem21 Group, Inc., 17870 Wilson Drive. Lake Forest, IL 60045, USA

**Keywords:** QSAR, 4D-QSAR, Structure-based QSAR, Drug Design

## Abstract

Drug design is a process driven by innovation and technological breakthroughs involving a combination of advanced experimental and computational methods. A broad variety of medicinal chemistry approaches can be used for the identification of hits, generation of leads, as well as to accelerate the optimization of leads into drug candidates. The quantitative structure–activity relationship (QSAR) formalisms are among the most important strategies that can be applied for the successful design new molecules. This review provides a comprehensive review on the evolution and current status of 4D-QSAR, highlighting present challenges and new opportunities in drug design.

## 1. Introduction

The identification of promising hits and the generation of high quality leads are crucial steps in the early stages of drug discovery process [1,2,3]. Advances in medicinal chemistry at the interface of chemistry and biology have created an important foundation in the search for new drug candidates possessing a combination of optimized pharmacodynamic and pharmacokinetic properties [4,5]. Drug discovery is currently driven by innovation and knowledge employing a combination of experimental and computational methods [6]. An understanding of the structure and function of the target, as well as the mechanism by which it interacts with potential drugs is crucial to this approach.

**Figure 1:**
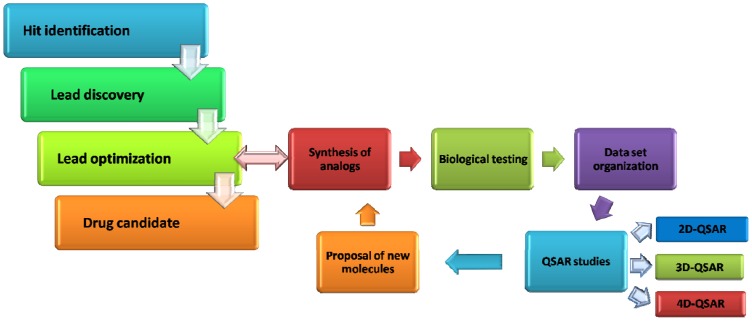
Schematic representation of the processes included in a lead optimization from the hit identification. QSAR methods are essential to reach this goal.

Quantitative structure-activity relationships (QSAR) play a vital role in modern drug design, since they represent a much cheaper and rapid alternative to the medium throughput in vitro and low throughput in vivo assays which are generally restricted to later in the discovery cascade. One would say that nowadays no drug is developed without previous QSAR analyses [7]. Figure 1 shows a flowchart of the process from hit identification to lead optimization, highlighting the important role of QSAR in drug design.

Origination of the modern QSAR formalism is attributed to the works of Hansch and Fujita [8] and Free and Wilson [9] in 1964. The QSAR methodology is based on the concept that the differences observed in the biological activity of a series of compounds can be quantitatively correlated with differences in their molecular structure [10]. Therefore, biological activity of congeneric molecular structures are related to specific molecular features (descriptors) by using regression techniques to estimate the relative importance of those features contributing to the biological effect. 

The classical QSAR methods [8,10] use as descriptors experimentally-derived molecular parameters (e.g., physicochemical data) and those calculated from the molecular connection table (2D structure). It is straightforward that experimental properties are a consequence of the entire three-dimensional structure (3D). However, they cannot be measured for non-synthesized compounds. On the other hand, the 2D descriptors, which can be calculated for idealized compounds, do not capture all of the information in the 3D structure [11,12]. Thus, when the study of the 3D molecular structure became practical routine with the parallel development of several computational molecular modeling techniques in the 1980s, the new era of the drug design process, named Computer-Aided/Assisted Drug Design (CADD) or Computer-Aided/Assisted Molecular Design (CAMD) [13] came into being and QSAR methodology has became in a broad subfield of CADD. Since then, several QSAR methodologies have been proposed. Each of them can be characterized by having particular approaches for calculating and selecting the molecular descriptors, and specific statistical algorithms for constructing the resulting models [11,12,14,15].

In analogy to the “direct” (*i.e.,* receptor-based, or structure-based) and “indirect” (*i.e.,* ligand-based) [16] approaches currently used in the CADD process, QSAR studies can be grouped in two major groups: receptor-independent (RI) and receptor dependent (RD) QSAR analyses [14]. In the first group either the geometry of the receptor is not available, or it is neglected in the QSAR analysis because of uncertainty in the receptor geometry and/or ligand binding mode. This group included the “classical” (zero-dimensional), one-dimensional (1D), two-dimensional (2D), three-dimensional (3D), and four-dimensional QSAR approaches [12]. The calculated descriptors are recognizable molecular features, such as atom and molecular counts, molecular weight, sum of atomic properties (0D-QSAR); fragment counts (1D-QSAR); topological descriptors (2D-QSAR); geometrical, atomic coordinates, or energy grid descriptors (3D-QSAR); and the combination of atomic coordinates and sampling of conformations (RI-4D-QSAR) [12]. In the RD-QSAR analysis, models are derived from the 3D structure of the multiple ligand-receptor complex conformations. This approach provides an explicit simulation of the induced-fit process, using the structure of the ligand-receptor complex, where both ligand and receptor are allowed to be completely flexible by the use of molecular dynamics (MD) simulation. RD-QSAR is used to gather binding interaction energies, as descriptors, from the interaction between the analog molecules and the receptor [7].

This review is intended to provide the reader with a brief overview of the current role of 4D-QSAR in drug design, highlighting the advances, challenges and future directions.

## 2. 4D-QSAR

As an evolution of Molecular Shape Analysis (MSA) [17,18], Hopfinger and co-workers proposed the 4D-QSAR formalism [19], which includes the conformational flexibility and the freedom of alignment by ensemble averaging in the conventional three dimensional descriptors found in traditional 3D-QSAR methods. Thus, the “fourth dimension” of the method is ensemble sampling the spatial features of the members of a training set. 

**Figure 2:**
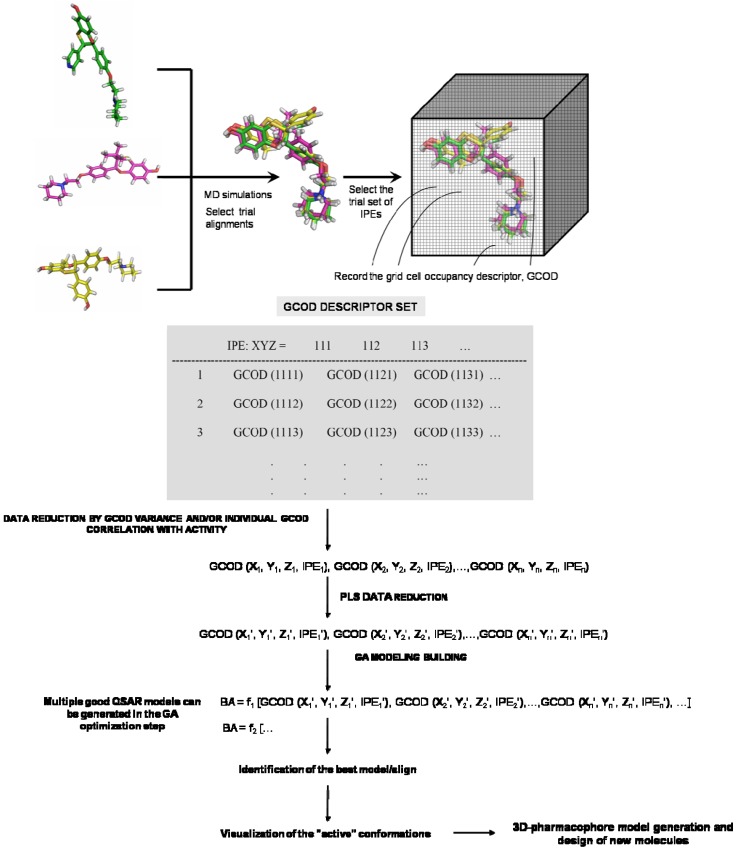
Schematic representation of the 4D-QSAR steps for the generation of models.

Figure 2 shows a scheme of the steps for the generation of 4D-QSAR models. In this approach, the descriptors are the occupancy frequencies of the different atom types in the cubic grid cells during the molecular dynamics simulation (MDS) time, according to each trial alignment, corresponding to an ensemble averaging of conformational behavior [20,21]. 

The grid cell occupancy descriptors, GCODs, are generated for a number of different atom types, called interaction pharmacophore elements, IPEs. These IPEs (*i.e.,* atom types), defined as “any type” (A or Any), “nonpolar” (NP), “polar-positive charge” (P+), “polar-negative charge” (P-), “hydrogen bond acceptor” (HA), “hydrogen bond donor” (HB), and “aromatic” (Ar), correspond to the interactions that may occur in the active site, and are related to the pharmacophore groups [19,22]. Thus, the IPEs are related to the descriptors’ nature in 4D-QSAR analysis, while the GCODs are related to the coordinates of IPE mapped in a common grid. The sampling process, in turn, allows the construction of optimized dynamic spatial QSAR models in the form of 3D pharmacophores, which are dependent on conformation, alignment, and pharmacophore grouping. 

The use of IPEs allows each of the compounds in a training set to be partitioned into sets of structure types and/or classes with respect to possible interactions with a common receptor. Sets of GCODs, defined by the IPEs, are simultaneously mapped into a common grid cell space. In the 4D-QSAR methodology a conformational ensemble profile of each compound is used to generate the independent variables (GCODs) instead of just one starting conformation. The variable selection is made using a genetic algorithm (GFA) [23].

One factor driving the development of 4D-QSAR analysis is the need to take into account multiple a) conformations, b) alignments, and c) substructure groups in constructing QSAR models. These “QSAR degrees of freedom” are normally held fixed in other 3D-QSAR analysis. 

In the CoMFA (Comparative Molecular Fields Analysis) [24] and GRID [25,26] formalisms the descriptors are calculated as grid point interactions between a probe atom and the target molecules and only one conformation of each compound is considered, not a conformational ensemble profile (as in 4D-QSAR method). They use different force fields, different types of probe atoms and the energy interactions are calculated differently. Interactions accounted for in the GRID force fields are steric (Lennard-Jones), electrostatic and hydrogen bonding interactions, and the total energy is the sum of all interactions. In contrast to CoMFA where the interaction energies (Lennard-Jones and electrostatic potentials) are considered separately, the sum of all the different interaction energies is calculated in each grid point with GRID [15,24,25]. The variable selection is made by the GOLPE (generating optimal linear PLS estimations) program [27], which is used also to perform the multivariate statistical analysis.

The CoMSIA (Comparative Molecular Similarity Indices Analysis) approach uses similarity measures between a probe atom (placed at each lattice position) and the molecules rather than CoMFA fields. Steric, electrostatic, and hydrophobic similarities are calculated using the SEAL program [28] to molecular superposition (similarity index).

Insofar as 4D-QSAR analysis can meaningfully predict “active” conformations and the preferred alignment for a training set, it may actually serve as a “preprocessor” for a subsequent CoMFA and/or CoMSIA. 

Furthermore, the 4D-QSAR method has been proven both useful and reliable for the construction of quantitative 3D pharmacophore models for ligand-receptor data sets [29,30,31,32,33].

## 3. Successful Applications of 4D-QSAR

The 4D-QSAR paradigm has been successfully applied in the construction of RI and RD-4D-QSAR models for a variety of enzyme inhibitors of different drug targets, such as HIV-1 protease [34,35], HIV-1 integrase [35], p38-mitogen-activated protein kinase (p38-MAPK) [36], 14-α-lanosterol demethylase (CYP51) [32], enoyl-ACP reductase from *M. tuberculosis* (InhA) [37], among others examples [30,31,33,34,38]. 

As a case study example, RI-4D-QSAR models were constructed for a set of thirty-four 5’-aryl-thiourea thymidine analogs, synthesized by Van Daele and co-workers [39], showing inhibitory activity against thymidine monophosphate kinase from *M. tuberculsosis* (TMPKmt). Details of the methodology used have been described elsewhere [29], as shown in Figure 2, but in short, the models were developed using 30 compounds (training set), and externally validated using four compounds (test set). The crystallized structure of deoxythymidine monophosphate (dTMP) co-crystallized with TMPKmt was retrieved from the Protein Data Bank (PDB entry code 1g3u) [40] and used for modeling all compounds. The 3D model for each structure was subsequently energy minimized, and submitted to AM1 semi-empirical calculation in order to obtain the partial atomic charges.

As noted above, the 4D-QSAR methodology can be used in a receptor-dependent, RD, mode when the geometry of the receptor is available as is the case here. However, RD-4D-QSAR analysis requires a relatively large and chemically diverse training set, and also definitive information on binding alignment(s), in order to achieve a non-ambiguous QSAR model. Unfortunately, these requirements were not met for this study and receptor independent, RI, 4D-QSAR analysis was carried out to maximize the extraction of structure-activity information. The benefits of doing the RI-4D-QSAR analysis performed as part of this study include;

a) providing a reliable and predictive 3D-pharmacophore model for the limited range of substituent sites and substituent chemistry;

b) developing a rational basis of where substituent can, and cannot, be placed on the scaffold structures of the analogs;

c) the use of the 3D-pharmacophore model as a docking alignment for general ligand-receptor modeling including future RD-4D-QSAR studies; and

d) employing the 4D-QSAR model as an initial virtual screening for future studies that can be structure-based.

**Figure 3:**
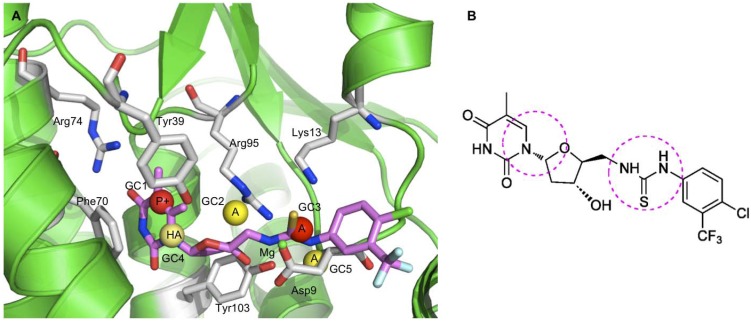
(**A**) Representation of the RI-4D-QSAR postulated “bioactive” conformation of the most potent inhibitor (**ATT14**) of the training set docked at the TMPKmt active site. Only the main interacting residues in the pocket of the binding site are shown in stick model representations (carbon atoms in gray). The inhibitor **ATT14 **is presented as stick models (carbon atoms in magenta). The GCODs of the best 4D-QSAR model are also shown in the active site of the crystal structure of TMPKmt, represented as spheres of 1 Å radius. Inhibition-enhancing and inhibition diminishing GCODs are shown, respectively, as yellow and red spheres. The IPEs atom types are as follows: A = any; NP = nonpolar; HA = hydrogen bond acceptor. (**B**) Chemical structure of **ATT14**, showing the main regions that contain pharmacophore sites, such as the sugar-pyrimidine ring structure and the 5′-arylthiourea moiety, which further can be explored to identify better inhibitors of TMPKmt.

The RI-4D-QSAR analysis [19,29] was carried out and the best 4D-QSAR model was graphically represented by plotting the significant grid cells in space along with their descriptor attributes (IPEs). The postulated “bioactive” conformation of the most potent inhibitor, according to the best 4D-QSAR model, was docked in the active site of the TMPKmt crystallographic structure (PDB entry code 1g3u) (Figure 3). There is a solid consistency between the 3D-pharmacophore sites defined by the QSAR models and interactions with binding site residues. Moreover, the model identifies new regions of the inhibitors that contain pharmacophore sites, such as the sugar-pyrimidine ring structure and the region of the 5′-arylthiourea moiety (Figure 3), that could be exploited to design new and more potent inhibitors of TMPKmt.

One of the two principal benefits of having done a 4D-QSAR analysis on this data set is in being able to identify and rank the relative importance of pharmacophore sites on the inhibitors that most influence the observed variance in inhibition potency. The other major benefit of this study is in being able to define a receptor alignment, using the 3D-pharmacophore sites of the 4D-QSAR equations, which provide a rational basis for ligand-receptor docking studies to be performed. 

The drawbacks to doing this 4D-QSAR analysis are largely a consequence of the relatively limited size and structural diversity of the data set used in the study. 4D-QSAR studies require greater size and diversity in training sets than other QSAR methods, especially 2D-QSAR approaches. This is because so many 4D-QSAR descriptors are created, the grid cell occupancy descriptors, all of which need to be reasonably sampled to build up reliable conformational occupancy profiles. The lack of analogs that sample all of the spaces around the scaffold structure[s], also limits the generality of a 4D-QSAR model. The model can only make structure-activity inferences for those spaces sampled by, in this case, the limited number of substituents at a limited number of the possible substitution sites on the scaffolds. 

One example of a successful application of the RD-4D-QSAR approach, was to a set of 48 4-hydroxy-5,6-dihydropyrone inhibitors of HIV-1 protease [41]. The receptor model used in this QSAR analysis was derived from the HIV-1 protease (PDB entry code 1d4s) crystal structure. The bound ligand in the active site of the enzyme, also a 4-hydroxy-5,6-dihydropyrone analogue, was used as the reference ligand for docking the data set compounds.

**Figure 4:**
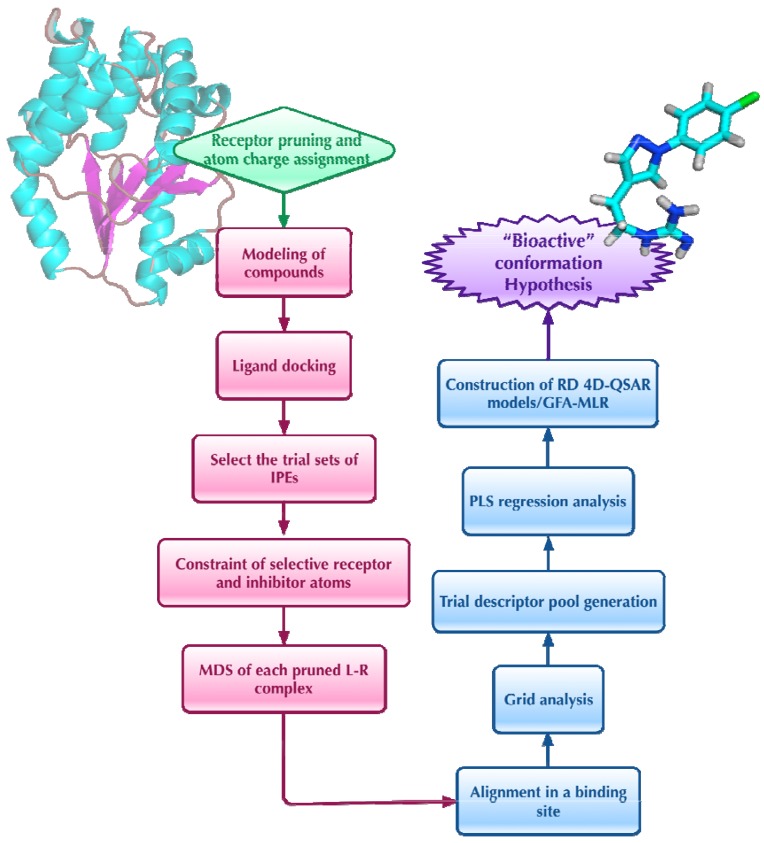
Operational steps in performing a RD-4D-QSAR analysis.

The RD-4D-QSAR analysis consists of 12 steps, which can be summarized as shown in Figure 4. A detailed description of the method is given in reference [42]. The main feature of a RD-4D-QSAR analysis is that the resultant pharmacophore sites of the QSAR models generated in the analysis are explicitly dependent upon the combined geometries of the (bound) ligand and the receptor. Moreover, the use of the 3D structure of the enzyme in constructing the 4D-QSAR models considerably improves the overall quality of the models.

The proposed “bioactive” conformations of the docked analogues into the active site of the HIV-1 protease were similar with those suggested from crystal structures. Moreover, the RD-4D-QSAR models also qualitatively “captured” the existence of specific induced-fit interactions between the enzyme active site and each specific inhibitor. Hydrophobic interactions, steric shape requirements, and hydrogen bonding of the 4-hydroxy-5,6-dihydropyrone analogues with the HIV-1 protease binding site model dominate the RD-4D-QSAR models in a manner, again, consistent with experimental conclusions. From the constructed models, it is possible to infer hypotheses for the development of new lead HIV-1 protease inhibitors [41]. The RD-4D-QSAR analysis of HIV-1 protease inhibitor SARs seems to provide a range of specific ligand-receptor binding information and, consequently, makes a considerable improvement over the previous corresponding RI-4D-QSAR analysis. First, the explicit use of the molecular geometry of the enzyme leads to very significant QSAR equations, as given by their *r^2^* and *q^2^* measures, and the corresponding test set predictions are remarkably good. There is also a propensity to be able to generate better models with fewer descriptor terms than those found for the corresponding RI-4D-QSAR models [41].

Although the 4D-QSAR method has traditionally been used to develop models with internal and external consistency, as well as predictive power, the same approach can be applied to estimate the activities of compound libraries, *i.e.* virtual screening (VS), for the identification of new hits [43]. The increasing demand for the analysis of large data sets such as those generated by combinatorial chemistry and high-throughput screening (HTS) techniques has demonstrated once again the versatility and range of applications of 4D-QSAR. Therefore, the 4D-QSAR models can be employed as a virtual high throughput screen, VHTS, in the analysis and design of virtual libraries [43,44]. The GCODS offer unique information to a VHTS, as they permit an assessment of how individual regions of space about a ligand contribute to the activity of the ligand in a screening assay. The activity (dependent variable) of each compound in the library can be estimated as a function of the number of GCODs in the 4D-QSAR model representing the VHTS. That is, multiple optimized 4D-QSAR models of increasing size [number of GCODs] can be used to carry out the virtual screening of a test compound. When increasing the size of the 4D-QSAR model yields no discernible change in the predicted activity, when compared to the prediction from the nearest smaller 4D-QSAR model, then one can conclude that the smaller 4D-QSAR model captures all of the pharmacophore sites for the test compound. The capacity to make this type of self-consistent estimate of activity in a VHTS is, we believe, unique to the 4D-QSAR methodology [44].

## 4. 4D-QSAR: Pharmacokinetic Studies and ADMET Prediction

In addition to unproved efficacy and toxicity, inadequate pharmacokinetic properties result in the withdrawal of a large proportion of drug leads from further development. Hence, key properties such as absorption, distribution, metabolism, excretion and toxicity (ADMET) have been recently considered in early phases of the drug discovery process [45,46]. This new paradigm has driven the need for large scale screening methods. *In vitro* and *in vivo* ADMET assays are lengthy, complex, and relatively expensive in terms of resources, reagents, and detection techniques. Recently, there has been a surge in computational efforts to estimate ADMET properties of drug-like compounds and a variety of useful *in silico* models has been developed with different levels of complexity, creating tools that are faster, simpler, and more cost-effective than traditional experimental procedures [45].

Blood-brain barrier (BBB) penetration is one of the most critical pharmacokinetic issues in the design of central nervous system (CNS) active drugs and a toxicity concern in the development of other classes of drugs and modeling BBB penetration is one major ADMET endpoint focus [47,48,49,50]. Efforts have particularly been made to construct a general BBB QSAR model from a large and structurally diverse training set [49,50].

The 4D-QSAR paradigm has been used to develop a formalism to estimate molecular similarity (MS) measures as a function of conformation, alignment, and atom type [49]. Molecular similarity can be measured in terms of the types of atoms composing each molecule leading to multiple measures of molecular similarity. This new method, using a combination of 4D-MS measures and cluster analysis to construct optimum QSAR models, was applied to a data set of 150 chemically diverse compounds to build optimum BBB penetration models [49]. The complete data set was divided into subsets based on 4D molecular similarity measures using cluster analysis. The compounds in each cluster subset were further divided into a training set and a test set. Predictive QSAR models were constructed for each cluster subset using the corresponding training sets. These QSAR models best predict test set compounds which were assigned to the same cluster subset, based on the 4D-molecular similarity measures, from which the models were derived. The results suggest that the specific properties governing BBB permeability may vary across chemically diverse compounds. Partitioning compounds into chemically similar classes is essential to constructing predictive BBB penetration models embedding the corresponding key physicochemical properties of a given chemical class.

## 5. 4D-Formalism: ‘Practical’ Application in Drug Design

The discovery of novel drug targets has increased exponentially in recent years due to advances in genomic and molecular biology techniques. Experimental and computational methods are effectively applied to accelerate the process of lead identification and optimization. HTS identifies lead molecules by performing individual biochemical assays with over millions of compounds, but it is huge cost and time consuming. These disadvantages have been overcome by the integration of cheaper and effective computational methodology as VHTS, which is widely applied to screen *in silico* collection of compound libraries to check the binding affinity of the target receptor with the library compounds. Accordingly the availability of structural data, VHTS is carried out using receptor-based or ligand-based screening methods. In both methods, the compounds are ranked using an appropriate scoring function regarding either complementarity or similarity and the top ranking compounds are taken to the next step of experimental assays [51]. Nowadays, the need for accurate and high-resolution virtual screening of the resulting hits is quite important and the development and use of VHTS with greater fidelity is becoming a goal in library design and evaluation.

Pharmacophore fingerprints are often used in the design and evaluation of compound libraries. A pharmacophore is generally a pattern of chemical groups in space that defines how ligands bind to a common receptor and also responsible for the biological response from the ligands. Additionally, the availability of the ligand at the site of action is related to its transport and metabolic behavior in the body environmental. Then, QSAR methods try to capture, or to extract, information about both the pharmacophore and availability components from a training set of compounds. The extent of pharmacophore and availability information that can be built into a QSAR model depends not only upon the training set, but also upon the descriptors used to represent them [52]. 

The 4D-fingerprints are descriptors derived from the 4D-MS methodology [53], which permits the generation of sets of molecular fingerprints that retain the conformational information of a compound as well as capture its size and chemical structure. Therefore, each molecular “finger” of the molecular fingerprint is specific to a particular atom/pharmacophore type present in a compound. 

A unique set of molecular fingerprints can be constructed for each specific alignment assigned to the compounds of a training set or library. So, alignment dependent molecular fingerprints permit molecular similarity measures to be developed as a function of the binding mode to a receptor site, for example. Otherwise, another unique set of molecular fingerprints in this class can be developed for any compound which are independent of alignment but do encompass the ensemble conformational states available. Methods that provide this kind of data are attractive for chemoinformatics, chemometrics, and molecular modeling applications. The respective 4D-fingerprints can be considered universal descriptors because they contain all the salient information about a compound [34]. One paper [34] reports the derivation and validation of a potential set of universal descriptors for generating descriptive QSAR models for five independent training sets. The models generated using the 4D-fingerprints were comparable in quality, based upon statistical measures of fit and test set prediction, to the previously reported models obtained using other QSAR methods.

## 6. Conclusions

Computational methods play a crucial role in modern medicinal chemistry, presenting a unique potential for transforming the early phases of drug research, particularly in terms of time and cost savings. Most of the techniques used in structure-based drug design have experienced significant improvements in the past few years, resulting in a remarkable enhancement of the speed and the efficacy of this approach. The successful application of 4D-QSAR models to generate 3D-pharmacophores of ligand-receptor data sets, to analyze and design of virtual libraries in a VHTS procedure and to predict ADMET properties has been described, showing that it can be a powerful tool in the early stages of drug discovery process. 

4D-QSAR analysis can also be applied to non-medicinal chemistry and biological problems. One such example in materials science is to predict how chelators will bind metal ions both in solution and on surfaces [44,54]. The practical applications are to design chelators that selectively remove specific ions from solutions and surfaces. Real world examples are keeping the walls of the tanks of hot water heater clean, swimming pool liners clean and making 'hard' water 'softer' by removing divalent ions like Ca^++^. 
